# Social networks moderate the association between physical fitness and cognitive function among community-dwelling older adults: a population-based study

**DOI:** 10.1186/s12877-021-02617-9

**Published:** 2021-12-07

**Authors:** Hui Foh Foong, Rahimah Ibrahim, Tengku Aizan Hamid, Sharifah Azizah Haron

**Affiliations:** 1 Malaysian Research Institute on Ageing (MyAgeing™), Universiti Putra Malaysia, 43400 Serdang, Selangor Malaysia; 2 Department of Human Development and Family Studies, Faculty of Human Ecology, Universiti Putra Malaysia, 43400 Serdang, Selangor Malaysia; 3 Department of Resource Management and Consumer Studies, Faculty of Human Ecology, Universiti Putra Malaysia, 43400 Serdang, Selangor Malaysia

**Keywords:** Cognitive function, Physical fitness, Social isolation, Social network

## Abstract

**Background:**

Physical fitness declines with age. Low levels of physical fitness appear to be a risk factor of cognitive impairment. Literature elucidates social networking as a potential moderator for the relationship between physical fitness and cognitive impairment. Present study aimed to examine the relationship between physical fitness and cognitive function among community-dwelling older Malaysians, and if social network moderates said relationship.

**Methods:**

Data of 2322 representative community-dwelling older adults were obtained from the first wave of the “Longitudinal Study on Neuroprotective Model for Healthy Longevity” national survey. Cognitive function, physical fitness and social network was assessed through Malay-version of Mini-Mental State Examination, 2-min step test and Lubben Social Network Scale-6 respectively. Moderated hierarchical multiple regression was employed to investigate if social networks moderate the relationship between physical fitness and cognitive function.

**Results:**

A positive association between physical fitness and cognitive function were found upon controlling for covariates. Moderated hierarchical multiple regression revealed social networks to be a moderator of the association between physical fitness and cognitive function. When physical fitness was low, those with small social network revealed lowest cognitive function.

**Conclusions:**

Social networks moderated the relationship between physical fitness and cognitive function as older adults with low levels of physical fitness and small social networks revealed lowest cognitive function. Therefore, community support or peer-based interventions among physically unfit older adults should be implemented to promote cognitive function.

## Background

Malaysia, like several other countries, is currently undergoing rapid population ageing. In 2016, 6.0% (1.9 million) of Malaysians were older adults aged 65 and above [1]. However, the number is set to rise to 15% (5.6 million) by 2035, at which point Malaysia will attain ageing nation status [2]. As such, prevalence of cognitive impairment is also expected to increase as most cognitive function domains tend to deteriorate with age, especially among women due to limited later life resources [3]. A recent study based on the Montreal’s Cognitive Assessment cut-off point of 22/23 found prevalence of cognitive impairment among Malaysian community-dwelling older adults aged 60 and above to be 75.2% [4].

Physical fitness refers to the body’s capacity to function effectively towards a healthy state, aside from its ability to perform daily and occupational activities. It has been established that physical fitness tends to decline with age. Poor physical fitness has also been highly correlated to older person morbidity and mortality. However, frequent exercise could help maintain physical fitness [5]. Physical fitness is an important determinant of cognitive function in old age, as evidenced by a recent study that found positive association between cognitive function and physical fitness in adults aged 55 and above [6]. Moreover, physical fitness was found to promote cognitive function among older patients with mild cognitive impairment [7].

Social networks, on the other hand refer to the web of social interactions and relationships, which apparently improves life satisfaction among older adults [8]. Older adults with limited social networks were at risk of social isolation [9]. In fact, approximately 49.8% of older Malaysians were at risk for social isolation [10], with significant associations found among women, non-Malays, non-homeowners, urban-dwellers, those living within larger household sizes, those with lesser number of siblings and those with lower perceived health status [10]. The role of social networks as a protective factor in development of cognitive impairment among older adults has been widely reported [11].

There are several grounds as to why social networks might act as a moderator in this study. Primarily, past literature has observed differing cognitive capacity and physical fitness levels among older adults with differing social network status. For instance, cognitive function was found to be higher among older adults with wider social network [12, 13]. Furthermore, a recent study discovered that social isolation among older adults were associated with daily physical inactivity [14], with reduced physical activity being the main contributor towards poor physical fitness among older adults [15]. In addition, decline in social networking among older adults were also attributed to gradual irreversible relinquishing of social roles, relegation of previous job roles, straying current social relationships, and collapse of extended family due to increasing age and retirement [16]. Considering the aforementioned reasons, this study aimed to examine the moderating role of social networks in the association between physical fitness and cognitive function to better understand the nature of interaction between social networks and physical fitness in predicting cognitive function. Three hypotheses were formulated in accordance to the study’s objective; H_A_1: Physical fitness was positively associated with cognitive function, H_A_2: Social networks were positively associated with cognitive function, and H_A_3: Social networks moderated the association between physical fitness and cognitive function.

## Methods

### Study design, participants, and procedure

The study involved secondary analysis of data sourced from Wave 1 (2012–13) of the “Longitudinal Study on Neuroprotective Model for Healthy Longevity” in Malaysia. Design of the study was cross-sectional, where data was collected from community-dwelling older persons aged 60 years or above through face to face, interviewer-administered surveys. Details of the study which includes sampling and research design have been formerly presented [17]. This survey utilized multi-stage stratified random sampling technique, beginning with random selection of a state from each zone in Peninsular Malaysia. After, 35 census clusters from each state were sampled, where 20 living quarters were selected from each census circle cluster. All respondents from the selected living quarters were randomly interviewed. Response rate obtained was 87.8%. Publications via similar methodology have been formerly presented [18]. The original dataset consisted of 2322 respondents and the present study involved all of them in the analysis without any exclusion criteria. The original study covered a wide range of variables; however, this paper only focused on cognitive function, physical fitness and social networks as per study interest. Authors established that all methods and procedures were performed in accordance with relevant guidelines and regulations.

### Measures

#### Cognitive function (dependent variable)

Cognitive function was evaluated through the Malay-version of Mini-Mental State Examination (MMSE) [19, 20]. The instrument included cognitive domains of visuospatial skills, language, concentration, working memory, memory recall, and orientation, assessed through 11 task items. Maximum obtainable score was 30, where higher score indicated higher cognitive ability. Cut-off point of 21/22 was apt for identifying older Malaysians with cognitive impairment [19]. MMSE scores served as a numeric discontinuous variable (score: 0-30) and as a categorical variable (cognitive impairment vs without cognitive impairment) for further analyses.

#### Physical fitness (independent variable)

The 2-min step was used to assess physical fitness [21]. Test validity have been formerly presented [22]. Respondents were first instructed to stand next to a wall, where the iliac crest and patella heights were measured and marked, respectively. After, a piece of tape was placed in between both marks. Respondents were then instructed to step in place by raising each knee towards the tape as many times as possible within 2 min. Physical fitness scores were calculated based on the number of times the right knee reached the required height. The cut-off point of scores was based on a functional fitness normative study among community-dwelling older adults by Rikli and Jones, with scores below 65 were associated with lower levels of functional ability [21]. The cut-off point was apt to identify the prevalence of older adults with lower levels of physical fitness [21]. Scores were also divided into two categories of fitness levels - lower levels (< 65) and higher levels (≥ 65). The 2-min step test scores served as a numeric discontinuous variable (number of times) and as a categorical variable (low fitness level vs high fitness level) for further analysis.

#### Social networks (moderator)

Social network was measured via Lubben Social Network Scale-6 [9]. This validated instrument evaluates social isolation among older adults by quantifying frequencies of social contact with friends and family members, as well as perceived social support obtained from stated sources. Obtainable scores ranged from 0 to 30, where higher scores indicated a larger social network. The cut-off point of 12 and lower were indicative of risk and prevalence for social isolation among older adults [9]. Social network scores obtained served as continuous and categorical variable for further analysis.

#### Covariates

The 4-step moderated hierarchical multiple linear regression was utilised to control effects of demographic variables like age, sex, year(s) of education, marital status, employment status, household income, ethnicity, comorbidity status, and living arrangement. Categorical variables were dummy-coded; sex (male = 0, female = 1), marital status (married = 0, not married = 1), employment status (currently employed = 0, currently not employed = 1), living arrangement (living with others = 0, living alone = 1), multimorbidity status (multimorbidity = 1, no multimorbidity = 0], and ethnicity (Chinese and Indian = 0, Malay = 1). Age, year(s) of education, and household income were measured in the continuous form.

### Statistical analysis

Statistical analyses were conducted via SPSS software (v23.0; IBM Corporation, Armonk, NY, USA). First, chi-square statistic was performed when the approach of the variables was categorical. Then, Pearson’s correlation, independent sample *t*-test, and moderated hierarchical multiple linear regression were performed when the approach of the variables was numerical. Associations between participant characteristics with key variables (i.e., physical fitness, cognitive function, and social networks) were first evaluated via chi-square statistics. Pearson’s correlation was then employed to observe bivariate correlations among the aforementioned key variables. Besides, independent sample *t*-test was used to observe differences in cognitive functions and social network between different physical fitness levels (i.e., high versus low). After that, a moderated hierarchical multiple linear regression was conducted to examine the moderating role of social network in the link between physical fitness and cognitive function. Control variables (i.e., sociodemographic and economic variables) were first added into the primary model. The independent variable (physical fitness) and moderator (social networks) were then entered into the secondary and tertiary model respectively. The final model involved addition of the interaction term (physical fitness × social networks). Significant *R*^*2*^ change indicated significant moderating effects. If the interaction term (physical fitness × social networks) is significant in the final model, the interaction graph is plotted to visualize the relationship between physical fitness and cognitive function based on three levels of social networks; − 1 standard deviation (SD) for small, 0 SD for moderate, and + 1 SD for large social networks. Continuous variables were centred prior to the regression analysis to prevent multicollinearity. VIF values obtained ranged from 1.010 to 1.510, less than 10, indicating non-presence of multicollinearity [23]. Statistical significance was set at two-sided *p*-value < 0.05.

## Results

### Background information of the sample

Table 1 depicts participants’ characteristics as well as distribution of respondents and study variables by levels of physical fitness level. The present study involved 2322 community-dwelling older adults, 51.9% of which were younger than 69 years old. Sex distribution was almost equal, where 52.2% of the respondents were women. Most were ethnic Malays (63.2%), married (60.9%), and living with others (89.3%). In terms of socioeconomic characteristics, majority received less than 7 years of education (76.4%), were not currently working (77.3%) and were from lower income group (91.1%). A total of 50.4% had a comorbidity status of more than one chronic condition. Demographic details have been formerly published [24].

**Table 1:** Respondents’ characteristics and distribution of study variables by levels of physical fitness

	Physical fitness			
	Higher levels of physical fitness (≥ 65 steps)	Lower levels of physical fitness (< 65 steps)	Chi-square statistic or *t-*value	*P*-value
	*n* (%) or mean ± standard deviation	*n* (%) or mean ± standard deviation		
Sex				
Men	599 (56.5)	461 (43.5)	88.82^a^	< 0.001^c^
Women	419 (36.5)	729 (63.5)		
Marital status				
Married	762 (50.4)	750 (49.6)	35.56^a^	< 0.001^c^
Not married	256 (36.8)	440 (63.2)		
Employment status				
Currently working	256 (51.4)	242 (48.6)	7.58^a^	0.006^c^
Currently not working	741 (44.4)	928 (55.6)		
Living arrangement				
Living with others	911 (46.1)	1067 (53.9)	0.02^a^	0.893^c^
Living alone	107 (46.5)	123 (53.5)		
Multimorbidity status				
No multimorbidity	527 (47.9)	574 (52.1)	2.74^a^	0.098^c^
Multimorbidity	491 (44.4)	616 (55.6)		
Ethnicity				
Malay	570 (41.3)	811 (58.7)	34.96^a^	< 0.001^c^
Non-Malay	446 (54.3)	376 (45.7)		
Cognitive function				
No cognitive impairment	752 (50.6)	733 (49.4)	39.64^a^	< 0.001^c^
Cognitive impairment	255 (36.3)	448 (63.7)		
Social networks				
No social isolation	578 (48.1)	623 (51.9)	5.33^a^	0.021^c^
At risk of social isolation	416 (43.2)	548 (56.8)		
Age	68.1 ± 5.58	69.5 ± 6.48	−5.88^b^	< 0.001^d^
Year(s) of education	6.1 ± 4.12	4.4 ± 3.69	10.52^b^	< 0.001^d^
Household income	1178.9 ± 1064.87	915.8 ± 940.54	5.97^b^	< 0.001^d^

^a^Chi-square statistic; ^b^*t*-value; ^c^Chi-square test; ^d^Independent sample *t*-test

Chi square statistic found significant associations between physical fitness and sex (*χ*^*2*^ = 88.82, *p* <  0.001), marital status (*χ*^*2*^ = 35.56, *p* <  0.001), employment status (*χ*^*2*^ = 7.58, *p* = 0.006), ethnicity (*χ*^*2*^ = 34.96, *p* <  0.001), cognitive function (*χ*^*2*^ = 39.64, *p* <  0.001), and social network (*χ*^*2*^ = 5.33, *p* = 0.021), respectively. Lower levels of physical fitness were prevalent among older adults whom were women, ethnic Malays, non-married, not currently working, cognitively impaired, and at risk of social isolation. Independent sample *t*-test reported significant differences in age (*t* = − 5.88, *p* <  0.001), year(s) of education (*t* = 10.52, *p* <  0.001), and household income (*t* = 5.97, *p* <  0.001) across different levels of physical fitness. Those within the lower levels of physical fitness had significantly lower education level, lower household income and older age (see Table 1). The use of the aforementioned cut-off points found prevalence of cognitive impairment, lower levels of physical fitness, and social isolation was 33.1, 54.1, and 40.0%, respectively.

### Correlations among main study variables and differences of cognitive function and social network by physical fitness level

The minimum, maximum, mean, and standard deviation of physical fitness, cognitive function, and social network are as reported in Table 2. Pearson’s correlation analysis stipulated a positive correlation between cognitive function, physical fitness (*r* = 0.17, *p* <  0.001) and social networks (*r* = 0.08, *p* <  0.001). Results also revealed physical fitness to be significantly and positively correlated with social networks (*r* = 0.10, *p* <  0.001) (see Table 1).

**Table 2:** Correlations among cognitive function, physical fitness, and social networks

Variable	Minimum	Maximum	Mean	SD	Correlation coefficient (*r*)		
					Cognitive function	Physical fitness	Social networks
Cognitive function	0	30	22.6	5.03	1		
Physical fitness	0	142	60.5	25.97	0.17***	1	
Social networks	0	30	13.7	6.55	0.08***	0.10***	1

*SD* Standard deviation

*** *p* <  0.001

Independent sample *t*-test was used to compare cognitive function and social network among respondents from different physical fitness groups. Older adults with lower physical fitness levels reported lower cognitive function in comparison to older adults with higher physical fitness levels (*t* = 6.714, *p* <  0.001). Similarly, those with higher levels of physical fitness also had significantly larger social networks (*t* = 3.090, *p* <  0.002) (see Table 3).

**Table 3:** Comparisons of cognitive function and social networks by different levels of physical fitness

Variable	Higher levels of physical fitness (≥ 65 steps)	Lower levels of physical fitness (< 65 steps)	*t*-value	*P*-value
	(mean ± SD)	(mean ± SD)		
Cognitive function	23.5 ± 4.64	22.1 ± 4.92	6.714	**< 0.001**
Social networks	14.2 ± 6.56	13.3 ± 6.54	3.090	**0.002**

Significance of bold values = *p* <  0.05

*SD* Standard deviation

### Moderating effects of social networks on the association between physical fitness and cognitive function

Four-step moderated hierarchical multiple regression was employed to examine the moderating role of social networks in the link between physical fitness and cognitive function. Confounding variables entered during the first model revealed a significant model [F (9,2040) = 39.345, *p* <  0.001, *R*^*2*^ = 0.148]. Sociodemographic and economic characteristics explained 14.8% of the variance in cognitive function. As shown in Table 4, younger age (*β* = − 0.11, *p* <  0.001) and higher educational levels (*β* = 0.27, *p* <  0.001) were associated with higher levels of cognitive function. Lower levels of cognitive function were also associated with women (*β* = − 0.07, *p* = 0.008) and ethnic Malays (*β* = − 0.15, *p* <  0.001).

**Table 4:** Associations between demographic variables, physical fitness, and social networks with cognitive function

Variable	Step 1^a^				Step 2^b^				Step 3^c^				Step 4^d^			
	B	SE	*β*	*p*-value	B	SE	*β*	*p*-value	B	SE	*β*	*p*-value	B	SE	*β*	*p*-value
Sex (0 = men, 1 = women)	− 0.63	0.24	− 0.07	**0.008**	−0.53	0.24	− 0.06	**0.028**	− 0.51	0.24	− 0.05	**0.034**	− 0.52	0.24	− 0.05	**0.031**
Age	− 0.09	0.02	−0.11	**< 0.001**	−0.08	0.02	−0.10	**< 0.001**	− 0.08	0.02	− 0.10	**< 0.001**	−0.08	0.02	−0.10	**< 0.001**
Year(s) of education	0.334	0.03	0.27	**< 0.001**	0.33	0.03	0.26	**< 0.001**	0.32	0.03	0.26	**< 0.001**	0.33	0.03	0.26	**< 0.001**
Marital status (0 = married, 1 = non married)	−0.05	0.26	− 0.01	0.847	− 0.05	0.26	− 0.01	0.863	− 0.04	0.26	− 0.01	0.880	− 0.04	0.26	− 0.01	0.872
Living arrangement (0 = living with others, 1 = living with others)	− 0.10	0.35	− 0.01	0.771	− 0.13	0.35	− 0.01	0.719	− 0.11	0.35	− 0.01	0.747	− 0.10	0.35	− 0.01	0.770
Household income	0.01	0.01	0.04	0.071	0.01	0.01	0.04	0.090	0.01	0.01	0.04	0.108	0.01	0.01	0.03	0.132
Ethnicity (0 = Chinese and Indian, 1 = Malay)	−1.48	0.21	−0.15	**< 0.001**	−1.43	0.21	−0.14	**< 0.001**	−1.46	0.21	−0.15	**< 0.001**	−1.47	0.21	−0.15	**< 0.001**
Multimorbidity status (0 = no multimorbidity, 1 = multimorbidity)	0.27	0.20	0.03	0.177	0.30	0.20	0.03	0.133	0.31	0.20	0.03	0.124	0.30	0.20	0.03	0.137
Employment status (0 = working, 1 = not working)	−0.21	0.25	−0.02	0.413	−0.19	0.25	−0.02	0.455	−0.17	0.25	−0.02	0.496	−0.16	0.25	−0.01	0.535
Physical fitness					0.01	0.01	0.05	**0.027**	0.01	0.01	0.05	**0.036**	0.03	0.01	0.15	**0.001**
Social networks									0.03	0.02	0.04	0.057	0.11	0.04	0.16	**0.002**
Interaction term (physical fitness × social networks)													−0.01	0.01	−0.17	**0.013**

Significance of bold values = *p* < 0.05

Model 1: F (9,2040) = 39.345, *p* < 0.001, *R*^2^ = 0.148, Sig F Change: *p* < 0.001

Model 2: F (1,2039) = 35.969, *p* < 0.001, *R*^2^ = 0.150, Δ*R*^2^ = 0.002, Sig F Change: *p* = 0.027

Model 3: F (1,2038) = 33.071, *p* < 0.001, *R*^2^ = 0.151, Δ*R*^2^ = 0.001, Sig F Change: *p* = 0.057

Model 4: F (1,2037) = 30.908, *p* < 0.001, *R*^2^ = 0.154, Δ*R*^2^ = 0.003, Sig F Change: *p* = 0.013

*B* Unstandardised coefficient, *SE* Standard error, *β* Standardised beta coefficient

^a^Demographic variables to predict cognitive function

^b^Demographic variables and physical fitness to predict cognitive function

^c^Demographic variables, physical fitness, and social networks to predict cognitive function

^d^Demographic variables, physical fitness, social networks and interaction term to predict cognitive function

The independent variable, physical fitness, was added in the secondary model, its addition significantly increasing model fit [F (1,2039) = 35.969, *p* <  0.001, *R*^*2*^ = 0.150, Δ*R*^2^ = 0.002]. As shown in Table 4, physical fitness was found to be positively associated with cognitive function (*β* = 0.05, *p* = 0.027). The addition of the moderator, social networks in the third model also significantly increased model fit [F (1,2038) = 33.071, *p* <  0.001, *R*^*2*^ = 0.151, *ΔR*^*2*^ = 0.001]. However, this study failed to establish a relationship between social networks and cognitive function, upon controlling for potential confounding factors (*β* = 0.04, *p* = 0.057) (see Table 4).

**Fig. 1:**
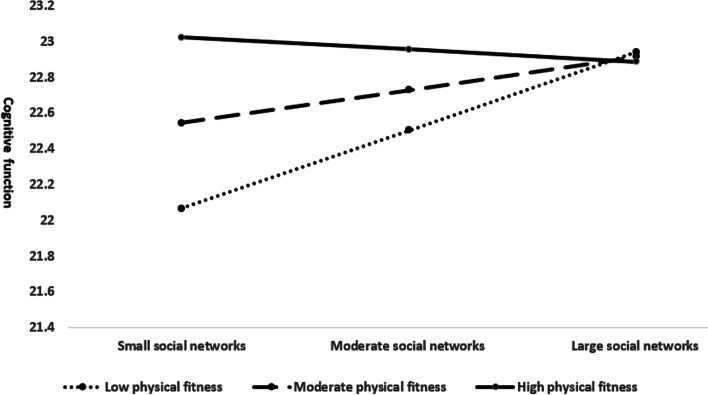
Interaction effect between physical fitness and social networks on cognitive function

The interaction term (physical fitness × social networks) was added into the last model, where a significant increase of model fit was observed [F (1,2037) = 30.908, *p* <  0.001, *R*^*2*^ = 0.154, *ΔR*^*2*^ = 0.003]. F change obtained was significant at *p* = 0.013 upon addition of the interaction term. As reported in Table 4, the interaction term was statistically significant (*β* = − 0.17, *p* = 0.013), hence indicating the moderating effect of social networks on the link between physical fitness and cognitive function. The relationship between physical fitness and cognitive function was plotted for small (− 1 standard deviation), moderate (0 standard deviation) and large social networks (+ 1 standard deviation) as shown in Fig. 1 to further observe the relationship at differing levels of social networks. As illustrated, older adults with low levels of physical fitness and small social networks revealed lowest cognitive function. Conversely, those with high levels of physical fitness and small social networks revealed highest cognitive function.

## Discussion

Findings from the study concurs that a positive correlation existed between physical fitness and cognitive function. However, there was no proof of an association between social networks and cognitive function after controlling for possible confounders, aside from the discovery that social networks moderated the association between physical fitness and cognitive function. H_A_1 was supported when the study revealed higher physical fitness levels were associated with higher cognitive function. Consistent with these findings are results from the Australia Stroke Prevention Study. The study found higher physical fitness in older persons to be associated with higher global cognitive function scores, memory, executive function, and motor abilities [25]. Several recent studies have also shown that physical activities promotes cognitive vitality [26, 27], Daimiel and colleagues argued based on their investigation involving 6874 participants in Spain, that physical fitness, but not physical activity, was associated with a higher cognitive function [28]. They found that physical fitness correlated with higher MMSE language domain score and total score [28]. Precise mechanism between physical fitness and cognitive function in older adults remains elusive. But proper cerebral perfusion and absence of cardiovascular diseases have been frequently proposed as possible mediators [29].

Findings revealed no support for H_A_2 as there was no link found between social networks and cognitive function upon controlling for confounders in the tertiary model. The non-significance found in this study cannot be deciphered, but it could probably be attributed to the way social network is measured and stronger relationships between certain sociodemographic variables (e.g., sex, age, ethnicity, and year(s) of education) with cognitive function. Nonetheless, the positive role of social networks towards cognitive function have been widely presented [30]. The positive relationship can be attributed to positive social networks which buffers stress, offers mental stimulation, and encourages health behaviours [31]. The current study evaluated just the quantitative elements of social networks like the functional and structural attributes of individual social network rather than exploring qualitative aspects of social networks such as perceived adequacy of and feelings related to social relationships. Literature have shown a positive association between qualitative characteristics of social networks with cognitive function among older adults [32]. Future studies should therefore consider measuring qualitative aspects of social networks alongside quantitative aspects.

However, this study was most keen on findings that revealed social networks as a moderator for the link between physical fitness and cognitive function, in the time supporting H_A_3. The study details those individuals with low physical fitness and small social networks showed poorest cognitive function. Therefore, indicating that the negative impact of low physical fitness on cognitive function seems to be more formidable among older adults with limited social networks. Wider old age social networks could reduce the negative impacts of low physical fitness towards cognitive function. A possible reason being larger social networks promote higher wellbeing and/or mental health [33]. Older adults with larger social networks are equipped with family members, relatives, and friends to confide in, especially in times of grave illness and loss, hence encouraging older adults to cope well and remain hopeful. A longitudinal study reported robust relationship between positive wellbeing and cognitive function [34]. Besides, the moderating role of social networks could also be attributed to different behaviours and activities due to different sizes of social networks. Older adults with larger social networks tend to have a healthier lifestyle and be physically active than those with smaller social networks [35], promoting a better cognitive function in later life. In other words, social networks have a moderating role in the relationship between physical fitness and cognitive function as a facilitator or inducer of behaviours and activities that promote higher physical fitness resulting in a higher cognitive function. This study also demonstrated that individuals with high levels of physical fitness reported high cognitive function despite small social network size. Therefore, implying that in place of quantitative aspects, qualitative aspects of social networks, such as relationship quality, contact frequency, and emotional closeness is imperative in maintaining cognitive function among older adults with high physical fitness.

The study acknowledges presence of several strengths and limitations. Firstly, the study involved a large and representative sample. Data obtained were representative of different sex, age groups and ethnicity. Thus, findings are generalizable to older adults living in Peninsular Malaysia. Further, the analysis process involved comprehensive control variables which produced reliable results, free from possible confounding effects. However, the study is a cross-sectional study, so causal conclusion is not warranted. Future studies should consider longitudinal data to validate findings from this study. Aside from that, the measure of physical fitness was limited to aerobic endurance. Other aspects of physical fitness, like muscular endurance, muscular strength, flexibility, and body composition were not included. Future studies should consider including the aforementioned aspects when measuring physical fitness. Finally, evaluation of social network was only focused on quantitative measures of social networks. Both quantitative and qualitative aspects of social networks could be a possible resource that promotes cognitive function among physically unfit older adults. Hence, it is suggested that future studies include qualitative aspect of social networks in order to obtain a clearer picture of domains influencing cognitive function among older persons.

## Conclusions

This study presents novel discovery on the moderating mechanism underpinning the relationship between physical fitness and cognitive function among older adults. Findings also adds to the literature elucidating the role of physical fitness and social network in predicting later-life cognitive function. This study found that social networks moderated the relationship between physical fitness and cognitive function as older adults with low levels of physical fitness and small social networks revealed lowest cognitive function. The moderating effects of social networks could be attributed to mental health, behaviours and activities as older adults with larger social networks tend to have better mental health, a healthier lifestyle and be more active. Hence, healthcare practitioners and volunteers are recommended to advance community support or peer-based interventions that encourage social networking among physically unfit community-dwelling older persons to prevent cognitive impairment and promote healthy ageing.

## Data Availability

The datasets used and/or analysed during the current study are available from the corresponding author on reasonable request.
